# Supplementary material for: Improved RNA stability estimation through Bayesian modeling reveals most Salmonella transcripts have subminute half-lives
Source: Proc Natl Acad Sci U S A. 2024 Mar 25;121(14):e2308814121. doi: 10.1073/pnas.2308814121 (PMC10998600; doi:10.1073/pnas.2308814121)
Supplement: Supplementary file 1 — Appendix 01 (PDF) [file pnas.2308814121.sapp.pdf]

PNAS

Supporting Information: Improved RNA stability estimation through Bayesian modeling reveals most bacterial transcripts have sub-minute half-lives

Laura Jenniches[1], Charlotte Michaux[2], Linda Popella[2], Sarah Reichardt[1], Jörg Vogel[1,2,3], Alexander J. Westermann[1,2], Lars Barquist[1,3,4,*]

[1] Helmholtz Institute for RNA-based Infection Research (HIRI), Helmholtz Centre for Infection Research (HZI), Würzburg, Germany
[2] University of Würzburg, Institute of Molecular Infection Biology (IMIB), Würzburg, Germany
[3] University of Würzburg, Faculty of Medicine, Würzburg, Germany
[4] Department of Biology, University of Toronto Mississauga, Mississauga, Ontario, Canada

*Correspondence to: lars.barquist@helmholtz-hiri.de

This PDF file includes:

Supporting Methods
Comparison between count-based and log-normal models
Tables S1 to S4
Figures S1 to S15
Supporting References

Other supporting materials for this manuscript include the following:

Dataset S1: Half-lives obtained by fitting the LNM to the RIF-seq data set.
Dataset S2: Steady-state log-fold changes of the RIF-seq data set from edgeR.
Dataset S3: Genetic features with ProQ-binding sites (CLIP-seq) in the 3’UTR or within 100 bases of the stop codon which are destabilized upon *proQ* deletion.
Dataset S4: Genetic features with CspC/E-binding sites (CLIP-seq) in the CDS or 5’UTR which are destabilized upon *cspC/E* deletion.
Dataset S5: Significant ProQ peaks obtained by re-analyzing the ProQ CLIP-seq data set (14).
Dataset S6: Significant CspC CLIP-seq peaks.
Dataset S7: Significant CspE CLIP-seq peaks.

Supporting Methods

Ratio between differential gene expression and stability in Δ*proQ* vs. Δ*cspCE*

The ratio between the differences in RNA half-life in the Δ*proQ* and Δ*cspCE* mutant strain (as compared to WT) was calculated by selecting only transcripts with significant stability changes in the same direction in the two RBP deletion strains (**Figure S9C**). Similarly, we selected only genes with significant log_2-fold changes in the same direction in both RBP deletion strains (**Figure 4F**).

Hydrogen peroxide exposure

Bacterial cultures of all strains (*Salmonella* WT, Δ*proQ*, *proQ*++ and Δ*oxyRS*) were grown overnight. All strains contain the pJV300 plasmid. 10 mL of culture were inoculated 1:1000 in LB and grown at 37°C for 5 h. The cultures were then diluted 1:100 in 10 mL of LB and incubated for 2 h at 37°C with 1.5 mM or 2 mM of H_2O_2. Controls were incubated without H_2O_2. Viability of the cells was assessed by spotting 5 µL of a dilution series (10^0, ..., 10^{-6}) on LB agar plates which were then incubated overnight at 37°C.

RNA secondary structure

The ViennaRNA web server with default settings was used to obtain the secondary structures of RNA sequences (1). Forna (2) was used for visualization.

Geneset enrichment analysis

In order to identify pathways with transcripts either stabilized or destabilized in the absence of ProQ or CspCE, the genes in the analysis were ranked according to the quantity $-\ sgn(\Delta t_{1/2}) log_{10}(p\ +\ 10^{-4}\ )$. For pathway analysis of log fold-changes, we used the quantity $-\ sgn(logFC) log_{10}(p)$ for ranking. We created a gene set database combining the terms for the strain SL1344 from the eggnog database (3), QuickGo (4) and KEGG (5). We used the r package GSEA 1.2 (6) to calculate the enrichment scores and the corresponding adjusted p values. gsea.type was set to 'preranked' and shuffling.type to 'gene.labels'. Gene sets with sizes between 3 and 50 genes were analyzed. For the CLIP-seq data, we performed a hypergeometric test with the R stats function fisher.test. The FDR corrected p value was obtained using the Benjamini Hochberg procedure. For the hypergeometric test, the significance cutoff on the CLIP-seq data was chosen as ($p_{adj} \leq 0.1$) for CsrA, Hfq, and ProQ. For CspC/E, the value was reduced to $p_{adj} \leq 0.01$ to obtain a comparable number of interaction partners.

RNase E cleavage sites in random sequences

To estimate expected overlap between CspC/E CLIP-seq peaks and RNase E cleavage sites (7), we generated 100 random peaks of the same length within the same transcript as the actual CspC/E CLIP-seq peak, then tested how many of these random peaks overlapped with RNase E cleavage sites. Across the 100 simulations, this resulted in a mean of 331 of overlapping

binding sites compared to 410 overlapping sites in the CspC/E CLIP-seq peaks. None of the 100 simulated sets yielded a value as high or higher than 410 overlapping binding sites, resulting in a p value of about 0.

UV Crosslinking, Immunoprecipitation, and RNA Purification

CspC/E CLIP-seq data sets were generated with the same protocol as (8, 9). In short, 400mL of bacterial culture was grown to an OD_{600} of 2.0 in three biological replicates. One half of the culture was irradiated with UV-C light at 800 mJ to induce RBP crosslinking. Cells were centrifuged and resuspended in lysis buffer, mixed with 1 ml glass beads and shaken for 10 minutes. Anti-FLAG magnetic beads were added to the lysate before rotating it for 1 hour at 4℃. The beads were collected by centrifugation, resuspended and subjected to multiple washing steps. Finally, the magnetic beads were collected on a magnetic separator and the supernatant was loaded and separated on a 15% SDS-polyacrylamide gel followed by transfer to a nitrocellulose membrane. The protein size marker was highlighted with a radioactively labeled marker pen, and the membrane was exposed to a phosphor screen for 30 min. The regions of the membrane containing radioactive signal were cut out, and the same regions were selected from the control samples (**Figure S7A**). The membrane pieces were cut into smaller pieces and incubated 1hr at 37℃ with shaking at 1000 rpm in a total volume of 400 µl of PK solution (200 µl of 2xPK buffer - 100mM Tris-Hcl pH 7.9; 10mM EDTA; 1% SDS - ;20 µl of Proteinase K (Fermentas, 20 mg/ml); 1 µl of SuperaseIN (Termo Fischer Scientist) completed with nuclease-free water up to 400 µl). After incubation, 100 µl of the PK solution containing 9M Urea was added to each tube and incubated for an additional 1hr at 37℃, 1000 rpm. For RNA extraction, phase-lock tubes (5PRIME) were used to mix 450 µl of Phenol:Chloroform:Isoamyl alcohol 25:24:1 (PCI; Roth) with the supernatant from proteinase K treated samples (around 450 µl). Phase lock tubes were incubated 5 min at 30℃ under agitation (1000rpm) and spined 15 min at 4℃, 13 000rpm. The aqueous phase was collected and precipitated using a 30:1 mix of 100% ethanol/3M Sodium Acetate pH 5.2 at -20℃ for at least 2hr. After 30min centrifugation at 4℃, 13 000rpm, the RNA pellets were washed with 70% ethanol and finally resuspended in 10 µl of nuclease-free water.

CLIP-seq cDNA Library Preparation and Sequencing

cDNA libraries were prepared using the NEBnext Multiplex Small RNA library kit (#E7300) according to the manufacturer’s recommendation. Briefly, for the 3’ SR adaptor ligation step, 2.5 µl of RNA sample extracted from CLIP elution was mixed with 1 µl of 3’SR adaptor, diluted 1:10 in nuclease-free water), incubate in thermal cycler 2min at 70℃. While on ice, a mix of 5 µl of 3' ligation reaction buffer and 1.5 µl 3’ ligation enzyme mix was added, and the samples were incubated for 1 hr at 25℃. For the RT primer hybridization, 2.75 µl of a 1:10 diluted SR RT primer was added to the samples following an incubation of 5 min at 75℃, 15 min at 37℃ and 15 min at 25℃. During the incubation period, 0.5 µl of a 1:20 5’ adaptor was incubated separately for 2 min at 70℃. This denatured 5’ adaptor was used for the 5’ SR adaptor ligation step where it was added to the samples with 0.5 µl of 10X 5’ ligation reaction buffer and 1.25 µl of 5’ ligation enzyme mix. The samples were then incubated for 1 hr at 25℃. For the final step, reverse transcription, to each sample was added 4 µl of first strand synthesis reaction buffer, 0.5 µl of murine RNase inhibitor and 0.5 µl of M-MuLV reverse transcriptase. The samples were

incubated for 1 hr at 50℃ and the RT enzyme later on inactivated at 70℃ for 15 min. For the cDNA amplification, 10 µl of each cDNA library was mixed with 25 µl of LongAmp Taq 2x Master mix, 1.2 µl of SR primer, 12.5 µl of nuclease free water and 1.2 µl of index primer (one different for each library). Amplification conditions applied were the following: 94℃ for 30 sec; 18 cycles of 94℃/15sec; 62℃/30sec; 70℃/15sec and a final step of 70℃ for 5 min. After amplification, samples were loaded of TBE gels and bands from amplification between 130 to 200 bp were selected by gel extraction. DNA was eluted from crushed gel pieces with 500 µl of DNA elution buffer after 2 hr incubation at RT. After collection of the supernatant using corning costar spin-X centrifuge tube filters, precipitation mix was added, and samples were placed at 80℃ for 1 hr. After centrifugation and washing steps, dried pellets were resuspended in nuclease free water. Size, quantity, and absence of primers dimers were checked by bioanalyzer before sequencing. High-throughput sequencing was performed by Vertis. The libraries were pooled on an Illumina Nextseq500 platform and sequencing done for single end 1x150 bp.

Processing of Sequence Reads and Mapping CLIP-seq

The CspC/E and ProQ CLIP-seq data (9) was analyzed following the procedure described in (8) with a few alterations. First, putative PCR duplicates were removed using FastUniq v1.1 (10). The read pairs were trimmed together using Cutadapt v4.1 (11) and reads with fewer than 12 remaining bases were discarded. Additionally, we performed quality trimming with a minimum phred score of 20. Read pairs longer than 25 nt were eliminated for peak calling. The remaining reads were mapped to the Salmonella Typhimurium SL1344 chromosome (NCBI Acc.-No: NC_016810.1) and plasmid (NCBI Acc.-No: NC_017718.1, NC_017719.1, NC_017720.1) reference sequences using segemehl version 0.3.4 (12) with an accuracy cutoff of 80%. Only uniquely mapping reads were considered for all subsequent analysis.For quantification of peak regions, no upper limit was imposed on read length. Reads were aligned to the Salmonella Typhimurium SL1344 chromosome and plasmids using STAR (13).

CLIP-seq Peak Calling

Segemehl read alignments were converted from BAM to BED format using BEDTools v2.17.0 and reformatted to satisfy blockbuster's input requirements. Subsequently, peaks were defined by applying blockbuster v0.0.1.1 (-minBlockHeight 10 -distance 1). This resulted in a large set of clusters with overlapping blocks of reads. In clusters with only one block the peak region was defined by the position of the block. In clusters with multiple blocks, peaks were chosen iteratively. First, the block with the highest count was selected and a peak region was defined by joining together all blocks which overlapped by at least 50% with this block. Then, all reads overlapping with this block were removed. This procedure was repeated until the largest block contained less than 1% of the reads in the corresponding cluster. A formalized description of this algorithm is given in (8).The peaks were exported to gff format and htseq-count v2.0.2 with default parameters was used to count the uniquely mapped reads in the STAR alignments.

Differential peak abundance analysis of CLIP-seq Data

DEseq2 (14) was used to identify peaks with differential abundance in the cross-linked vs. the non-cross-linked libraries. Log-fold changes were shrunk using apeglm (15). We required a log-fold change of at least 1. For ProQ, we chose the same adjusted p-value cutoff as (8) chose

for CsrA and Hfq (padj<0.1, **Figure S7B**). For the CSPs, the adjusted p-value cutoff was reduced to 0.01 to obtain a comparable number of peaks (**Figure S7C**).

Comparison between count-based and log-normal models

The modeling of RNA-seq count data conventionally employs a negative binomial (NB) distribution, as demonstrated by tools such as edgeR(16) and DESeq2(14). Notably, the authors of the limma package(17) have previously shown that modeling log-transformed counts with normal distributions yields comparable (or even superior) results to count-based models in simulations where data was explicitly generated from a negative binomial distribution, provided that measurement precision is accounted for. Given the similarity of our RIF-seq library sizes (Figure S2M) and, consequently, measurement precision across replicates, we did not explicitly model measurement precision differences in our LNM model.

To validate this choice, we conducted a comparative analysis between the LNM, a count-based NB model, and a log-normal model with count-dependent variance (LNMcdv), similar to the limma-voom package(17). Our investigation revealed a very high correlation between wild-type decay rates, differences in decay rates, and p-values obtained from our original LNM and the two alternative models (NB, LNMcdv, see **Figure S15A-D**). Furthermore, we confirmed that the results are still highly similar when working with a reduced dataset (replicates 1,2 and 3 of the ProQ dataset, **Figure S15E**). However, the negative binomial model in particular came with significant computational costs, requiring between 3 and 4 fold more CPU time for sampling (**Figure S15F**). Considering the computational efficiency and the consistent results obtained with the three models, the application of the simple LNM for our RIF-seq dataset is well-founded.

Supporting Figures

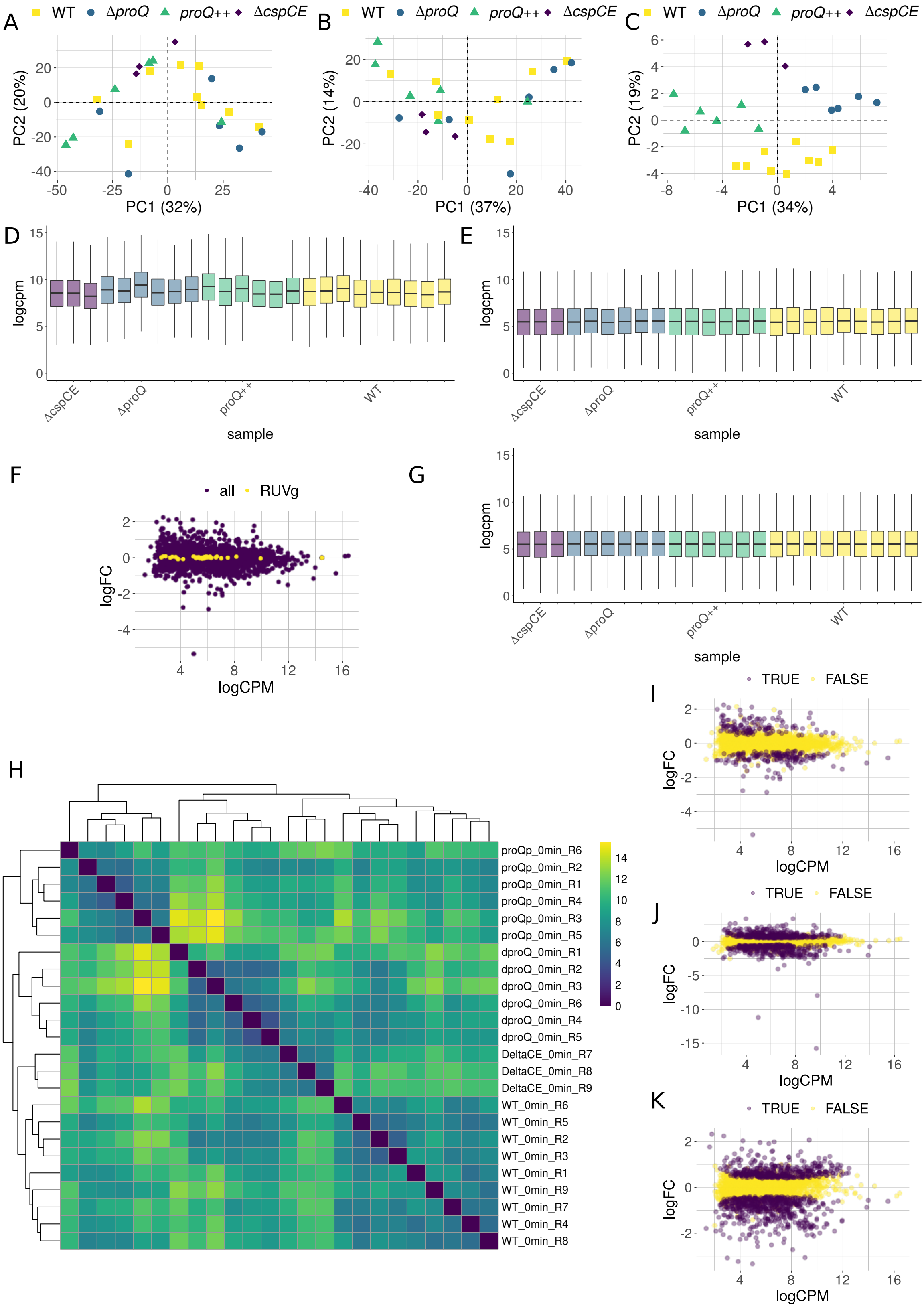

Figure S1. Differential expression (DE) analysis at t = 0 **min**
(A) PCA plot after normalizing by library size. (B) PCA plot after TMM normalization. (C) PCA plot after running RUVg. (D) logcpm values after normalizing by library size. (E) logcpm values after TMM normalization. (F) MA plot ΔproQvs. WT after TMM normalization. Genes used in RUVg are marked in yellow. (G) logcpm values after running RUVg. (H) Samples clustered by euclidean distance after running RUVg. (I-K) MA plots for ΔproQ , proQ++, and ΔcspCE (top to bottom) vs. WT after normalization with RUVg. Significantly DE genes (FDR $<$ 0.05) are highlighted.

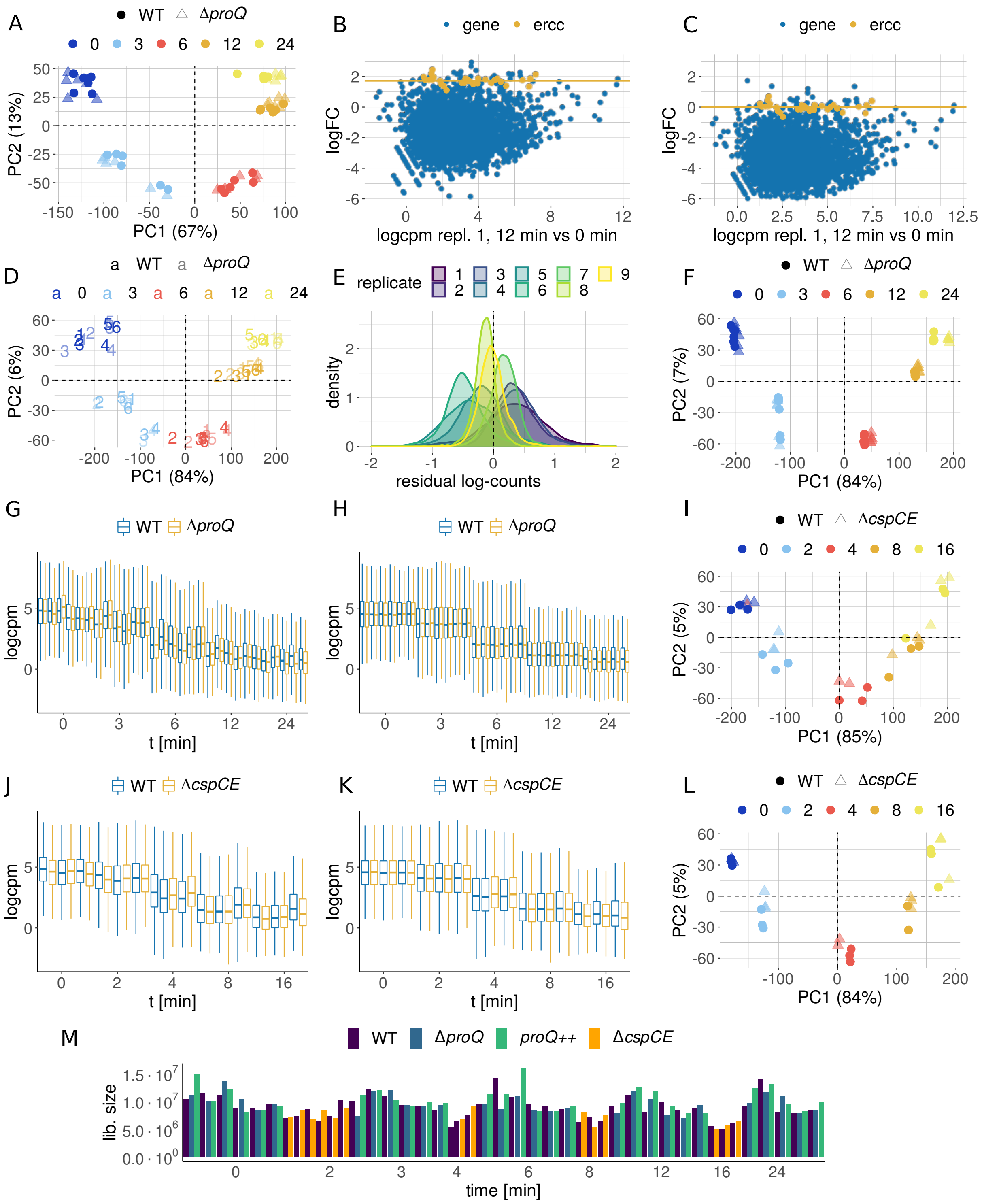

Figure S2. Normalization

(A) PCA plot of the raw proQ RIF-seq data. (B) Representative MA plot before TMM normalization with ERCC spike-ins. (C) Representative MA plot after TMM normalization with ERCC spike-ins. (D) PCA plot after normalization with ERCC spike-ins. (E) Illustration of the CM normalization for WT at min: After subtracting the condition-wise mean, the offset of the mean from 0 provides an additional normalization constant. (F) PCA plot after center-mean (CM) normalization. (G) Relative log-expression (RLE) of WT and ΔproQ libraries after TMM normalization. (H) RLE of WT and ΔproQ libraries after CM normalization. (I) PCA plot after TMM normalization with ERCC spike-ins. (J) RLE of WT and ΔcspCE libraries after TMM normalization. (K) RLE of WT and ΔcspCE libraries after CM normalization. (L) PCA plot after center-mean (CM) normalization. (M) Total number of reads (library size) across all samples.

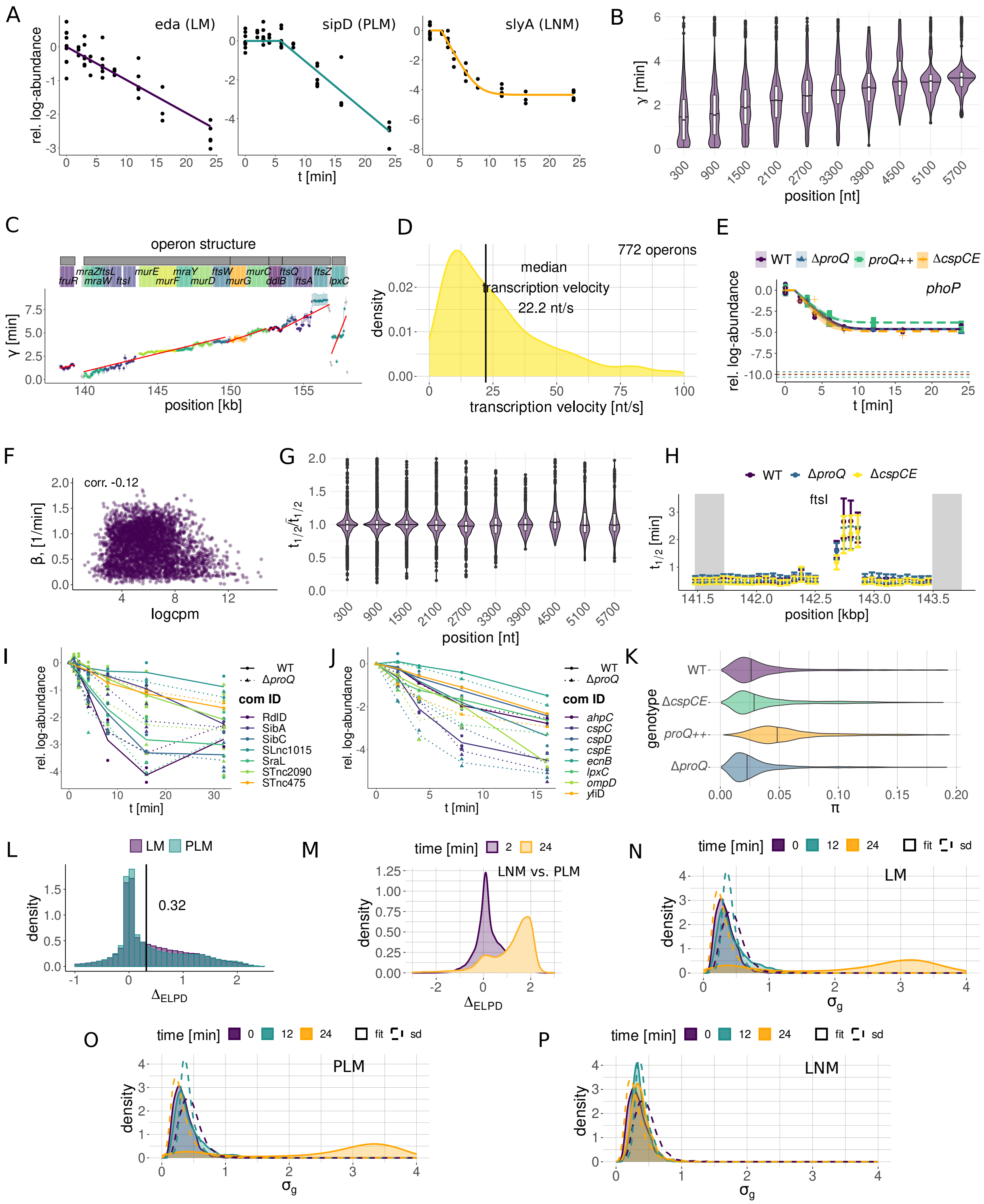

Figure S3. Model development, global transcript stability and transcription rate
(A) Representative WT decay curves exhibiting the dynamics correctly described by either the LM, PLM or LNM. (B) Genome-wide association of elongation time γ with the annotated primary transcription start sites. (C) An example of how the transcription rate was extracted from the elongation times of the 60 base windows including annotations and operon structure. Primary TSSs are indicated by black lines. (D) Distribution of genome-wide transcription rates as extracted from the 60 base windows.

(E) Comparison of decay curves and detection limit due to adding a pseudocount for the phoP transcript. (F) Scatter plot of gene expression and decay rate. (G Half-lives of the 60 base windows scaled to the gene-average relative to the start of the CDS. (H) Stable decay intermediate in the CDS of the ftsI mRNA which corresponds to the sRNA FtsO. FtsO (also called STnc475) has previously been shown to be destabilized in the absence of ProQ (19). (I) Northern blot quantifications of sRNA from (19). (J) Northern blot quantifications of mRNA from (9). (K) Distribution of genome-wide stable baseline fraction ordered by genotype. (L) Difference between ELPD of the LNM and the LM/PLM, respectively. Positive values favor the LNM. (M) Difference in ELPD between the LNM and the PLM at 24 min. Positive values favor the LNM. (N) Fitted unexplained variation $\sigma_g(t)$ in the LM compared to calculated standard deviation. (O) Fitted unexplained variation $\sigma_g(t)$ in the PLM compared to calculated standard deviation. (P) Fitted unexplained variation $\sigma_g(t)$ in the LNM compared to calculated standard deviation.

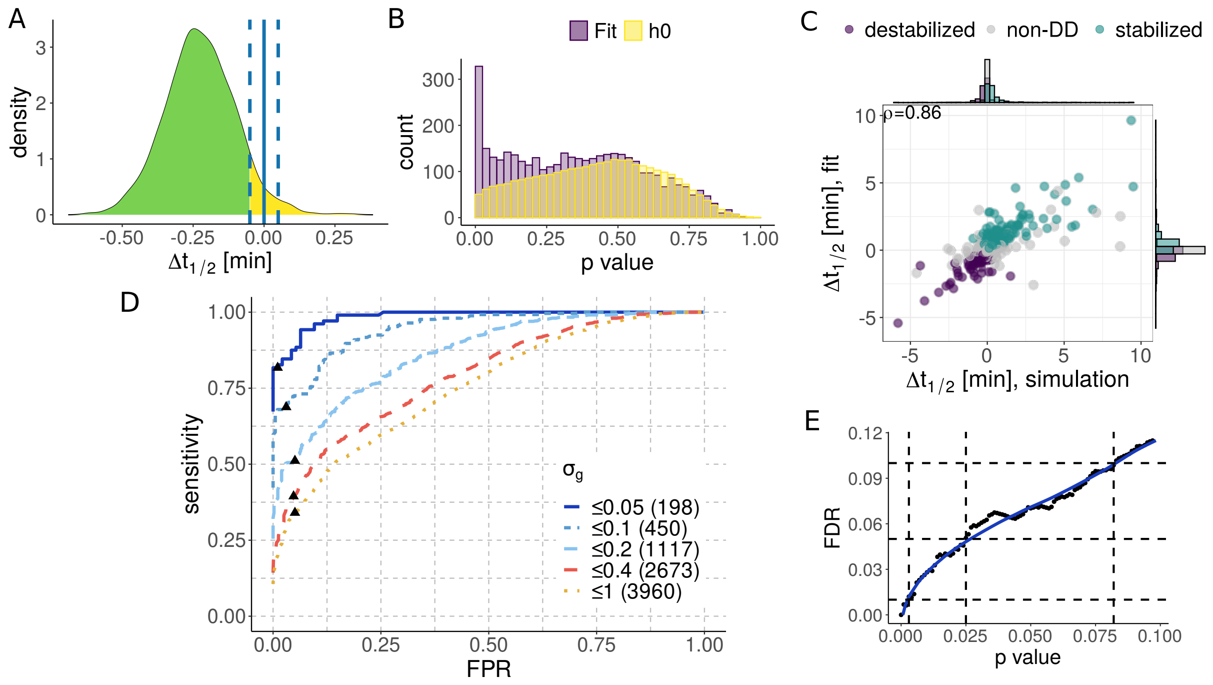

Figure S4. Posterior predictive p values und false discovery rate (FDR)
(A) Posterior distribution of the difference in half-life for a transcript in the RBP deletion strain vs. the WT. Under the null hypothesis, MCMC samples should fall within an interval around zero (blue, dashed lines). The Bayesian p value is given by the fraction of samples that overlap with the null hypothesis (yellow). (B) p-value distribution for differential stability data for the ΔproQstrain compared to the distribution under the null hypothesis. (C) Correlation between simulated and fitted differences in half-life (Pearson $\rho = 0.86$). (D) ROC curves obtained from the simulated data for different simulated standard deviations of relative log-counts. An FDR of 0.1 is marked with a black triangle. The number of transcripts which pass the cutoff is indicated in parentheses. At an FDR of 0.1, differentially decaying transcripts with a simulated standard deviation below 0.05 are identified with a sensitivity of 0.82. (E) We determine the FDR at a given p value cutoff from the simulated data (black dots) and fit a LOESS curve to it to map p-values to FDR (blue line). More details on the calibration of p values can be found in the Methods.

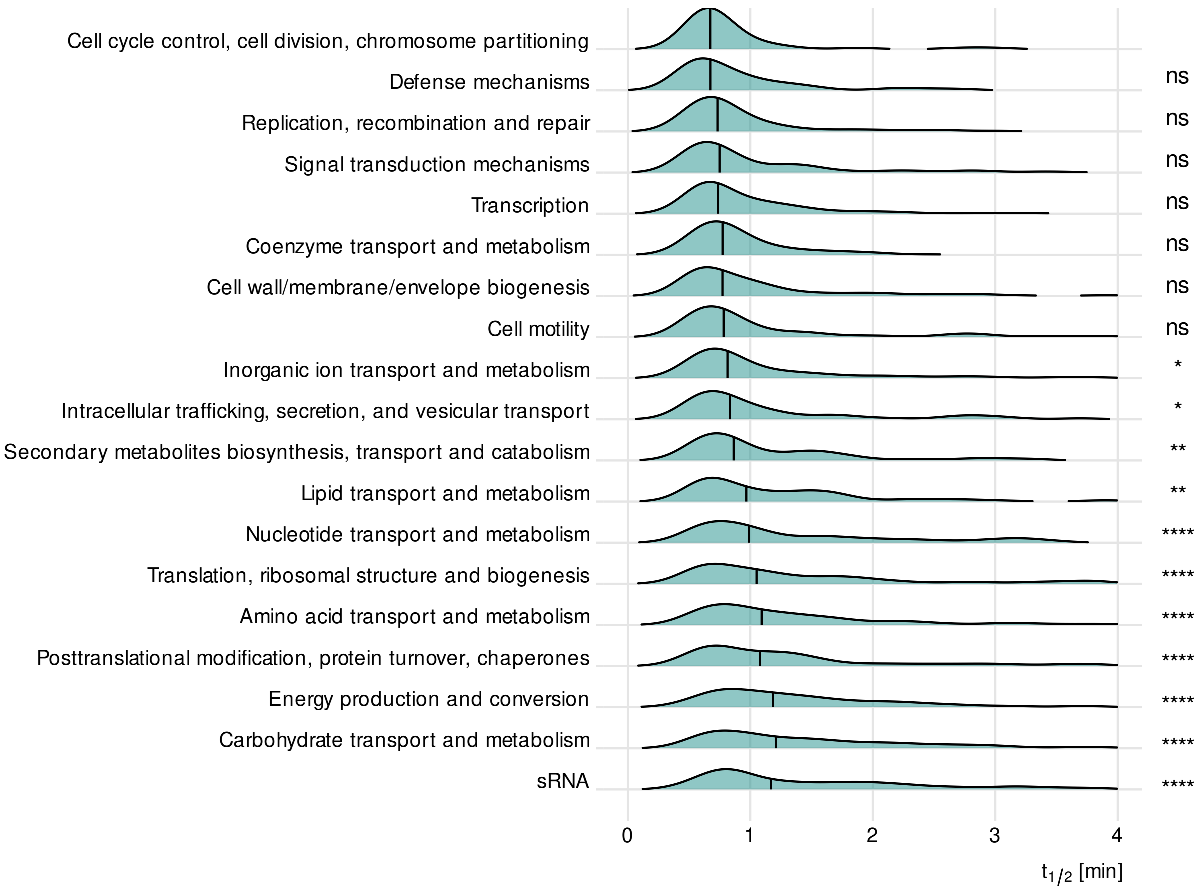

Figure S5. Median half-lives for transcripts classified by COG category. Related to Figure 1F. Adjusted p values (compared to the first COG) were calculated using the Wilcoxon rank sum test.

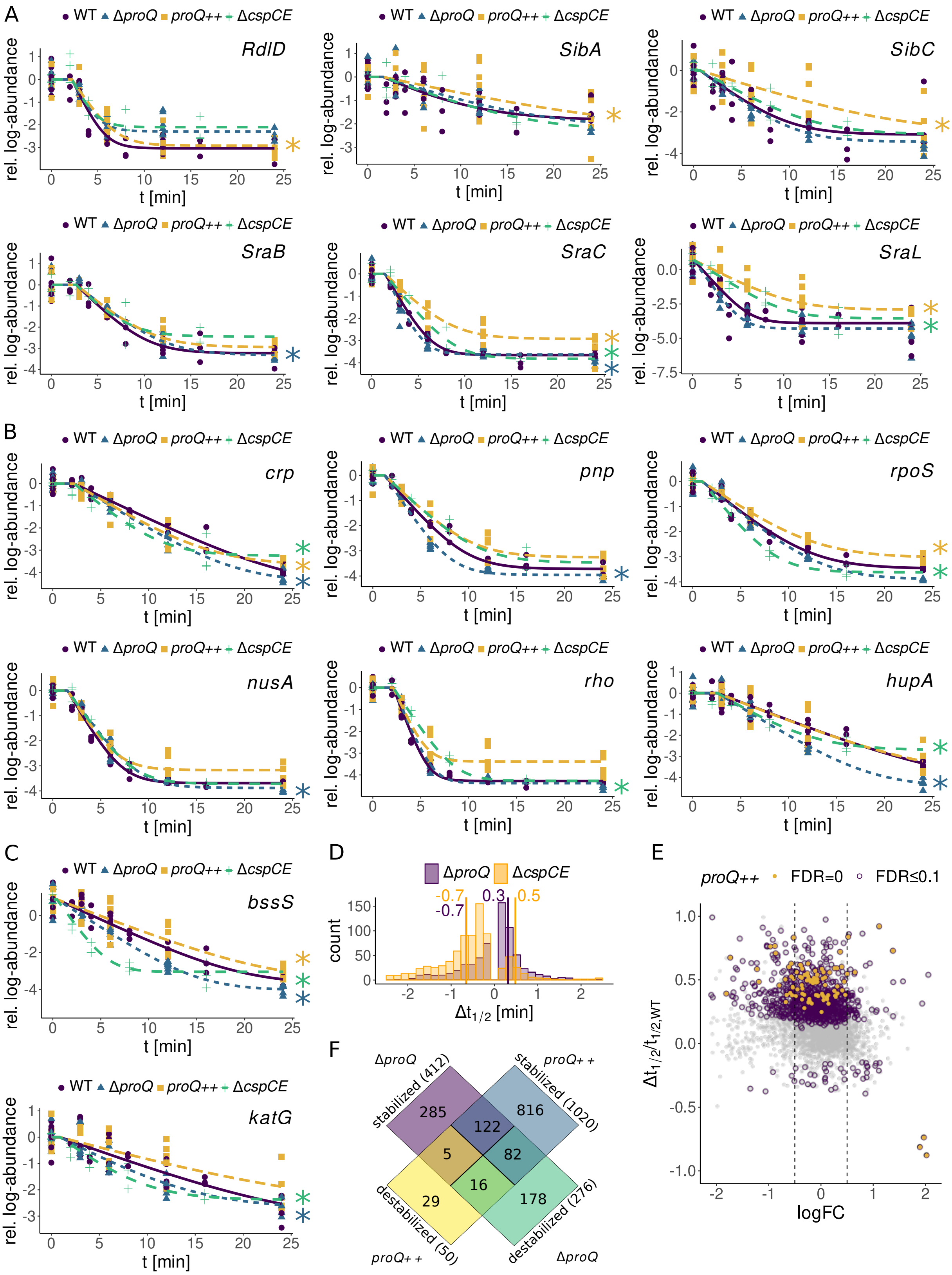
A
WT ΔproQ proQ++ ΔcspCE
RdlD
SibA
SibC
SraB
SraC
SraL
B
crp
pnp
rpoS
nusA
rho
hupA
C
bssS
katG
rel. log-abundance
t [min]
D
ΔproQ ΔcspCE
-0.7
-0.7
0.3
0.5
count
Δt1/2 [min]
E
proQ++ FDR=0 FDR≤0.1
Δt1/2/t1/2,WT
logFC
F
ΔproQ
stabilized (412)
proQ++
stabilized (1020)
285
122
816
5
82
16
29
178
destabilized (50)
destabilized (276)
proQ++
ΔproQ

Figure S6. Representative RIF-seq results (A) Stability changes for known ProQ sRNA targets. Significant stability changes are marked with a star. Stability changes upon proQ deletion were not significant, but in agreement with previously published results (19), all except for SraB were hyperstabilized upon proQ overexpression. (B) Stability changes for the exoribonuclease PNPase and global transcriptional regulators. Significant stability changes are marked with a star. (C) Transcripts with large stability changes upon deletion of proQ and cspCE. Significant stability changes are marked with a star. (D) Distribution of significant stability changes in the two RBP deletion mutants. (E) Relative difference in half-life vs. steady state log-fold changes between the proQ++ and the WT strain. (F) Overlap between transcript stability changes upon proQ deletion or overexpression.

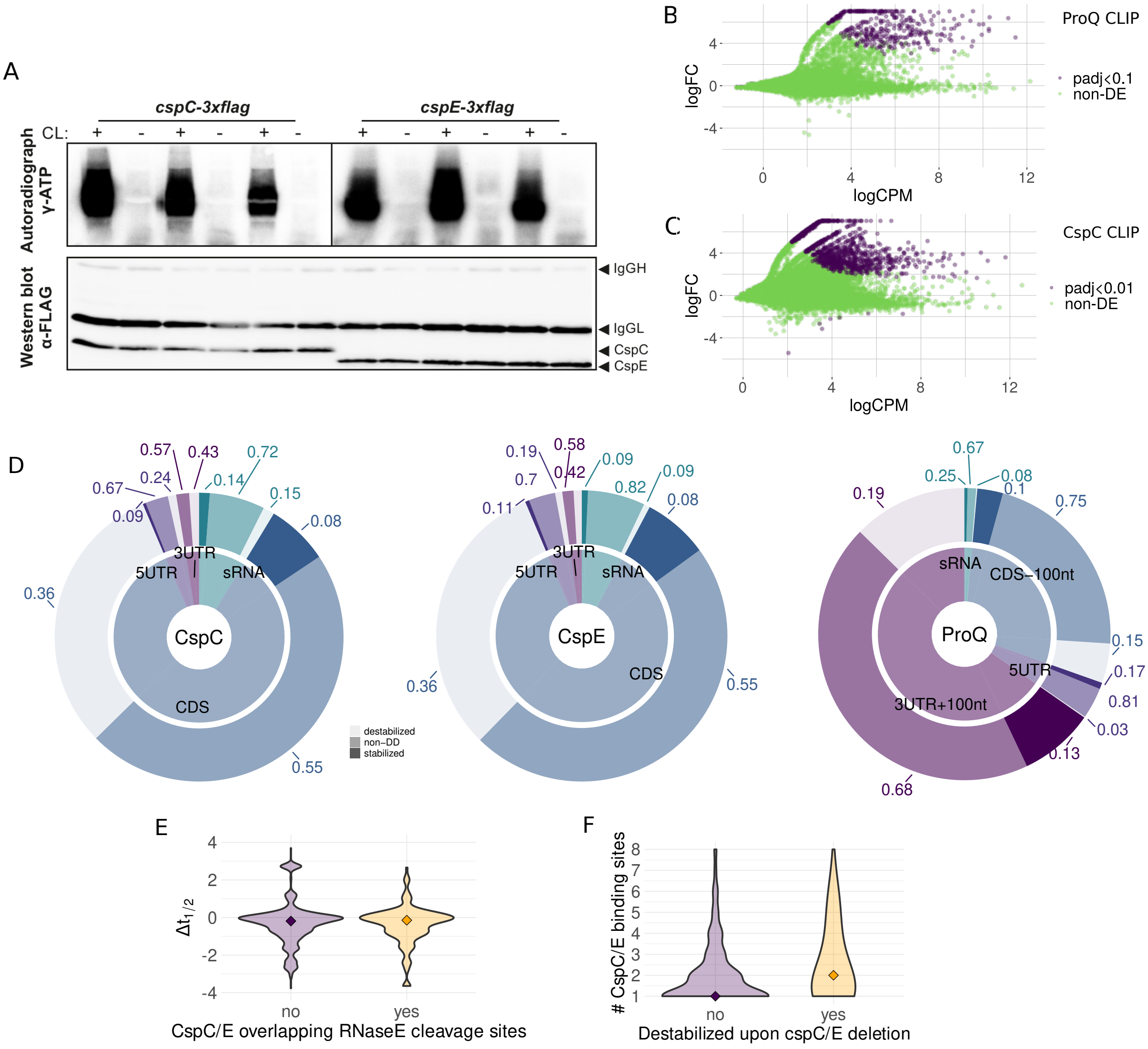

Figure S7. CLIP-seq results and comparison to RIF-seq

(A) Autoradiograph of radioactively labeled RNA fragments covalently bound by CspC/E after UV cross-linking (CL), immunoprecipitation, gel electrophoresis, and membrane transfer. The presence of the RBPs was verified by western blotting. (B,C) MA plots of the CLIP-seq analysis. (D) Fraction of transcripts bound by CspC/E/ProQ which are (de-)stabilized upon cspC/E deletion or which do not decay differentially (non-DD). Peaks which overlap with the CDS and the UTR have been assigned to the CDS only. For ProQ, the region within 100 nt of the stop codon has been analyzed jointly with the 3'UTR. (E) Changes in transcript stability of CspC/E targets categorized by whether or not the binding site overlaps a known RNase E cleavage site. (F) Number of CspC/E binding sites per transcripts categorized by whether or not the transcript is destabilized upon cspC/E deletion. Wilcoxon rank sum test: p = 0.000115.

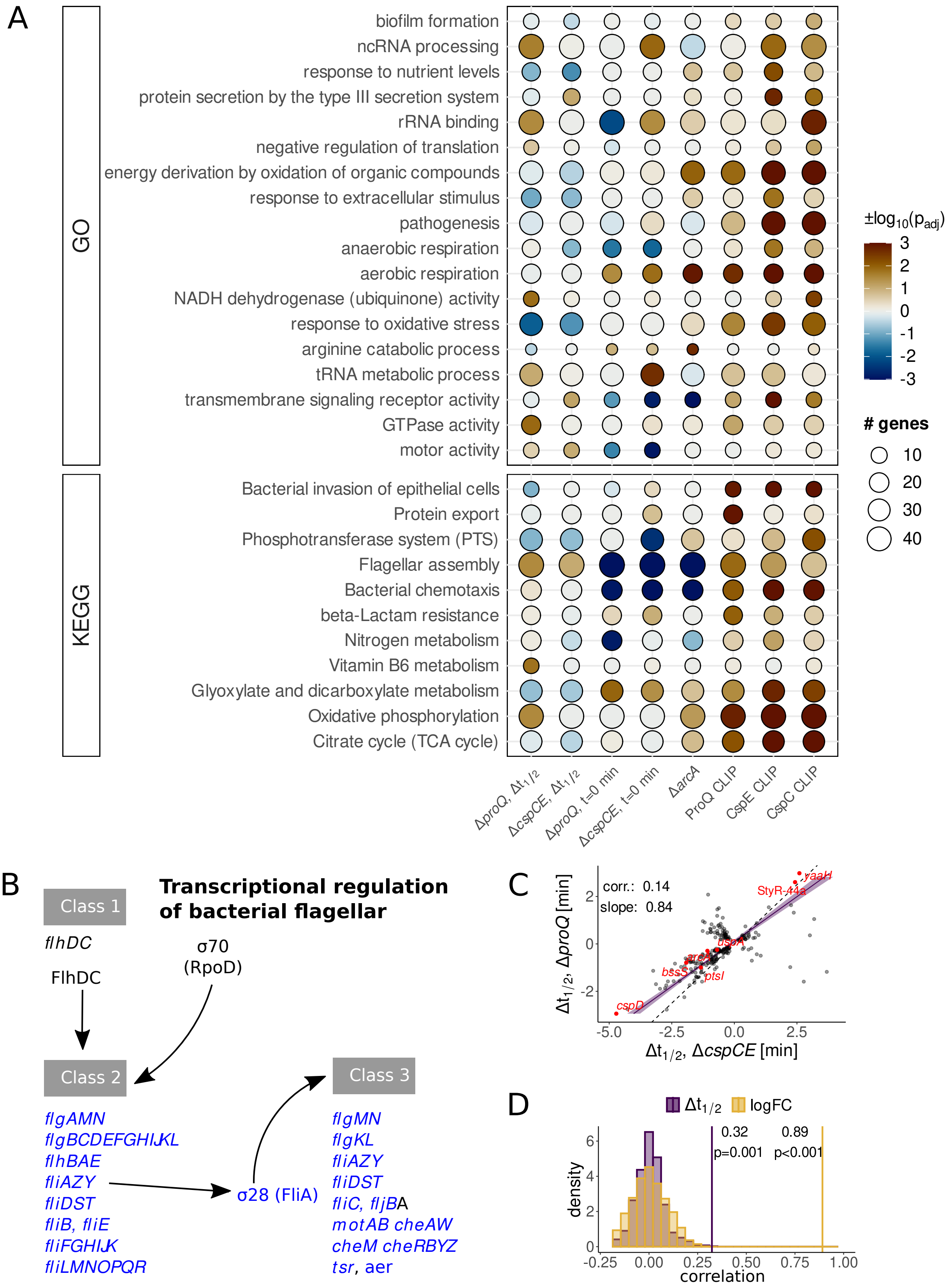
A
GO
KEGG
biofilm formation
ncRNA processing
response to nutrient levels
protein secretion by the type III secretion system
rRNA binding
negative regulation of translation
energy derivation by oxidation of organic compounds
response to extracellular stimulus
pathogenesis
anaerobic respiration
aerobic respiration
NADH dehydrogenase (ubiquinone) activity
response to oxidative stress
arginine catabolic process
tRNA metabolic process
transmembrane signaling receptor activity
GTPase activity
motor activity
Bacterial invasion of epithelial cells
Protein export
Phosphotransferase system (PTS)
Flagellar assembly
Bacterial chemotaxis
beta-Lactam resistance
Nitrogen metabolism
Vitamin B6 metabolism
Glyoxylate and dicarboxylate metabolism
Oxidative phosphorylation
Citrate cycle (TCA cycle)
ΔproQ, Δt1/2
ΔcspCE, Δt1/2
ΔproQ, t=0 min
ΔcspCE, t=0 min
ΔarcA
ProQ CLIP
CspE CLIP
CspC CLIP
±log10(padj)
3
2
1
0
-1
-2
-3
genes
10
20
30
40
B
Class 1
Transcriptional regulation of bacterial flagellar
flhDC
FlhDC
σ70 (RpoD)
Class 2
Class 3
flgAMN
flgBCDEFGHIJKL
flhBAE
fliAZY
fliDST
fliB, fliE
fliFGHIJK
fliLMNOPQR
σ28 (FliA)
flgMN
flgKL
fliAZY
fliDST
fliC, fljBA
motAB cheAW
cheM cheRBYZ
tsr, aer
C
corr.: 0.14
slope: 0.84
yaaH
StyR-44a
uspA
areA
bssS
ptsI
cspD
Δt1/2, ΔproQ [min]
Δt1/2, ΔcspCE [min]
D
Δt1/2
logFC
0.32
p=0.001
0.89
p<0.001
density
correlation

Figure S8. Pathway analyses

(A) Summary of pathway analyses. (B) Transcriptional changes of flagellar genes. Genes highlighted in blue are downregulated upon both proQ and cspC/E deletion (see Figure S12 for more details). (C) Correlation between differences in half-life in the ΔproQ and ΔcspCE strains. Only transcripts significantly different in both deletion mutants were considered. The linear regression includes only transcripts with changes in the same direction. (D) Significance of correlation between abundance and stability changes in the ΔproQ and ΔcspCE strains.

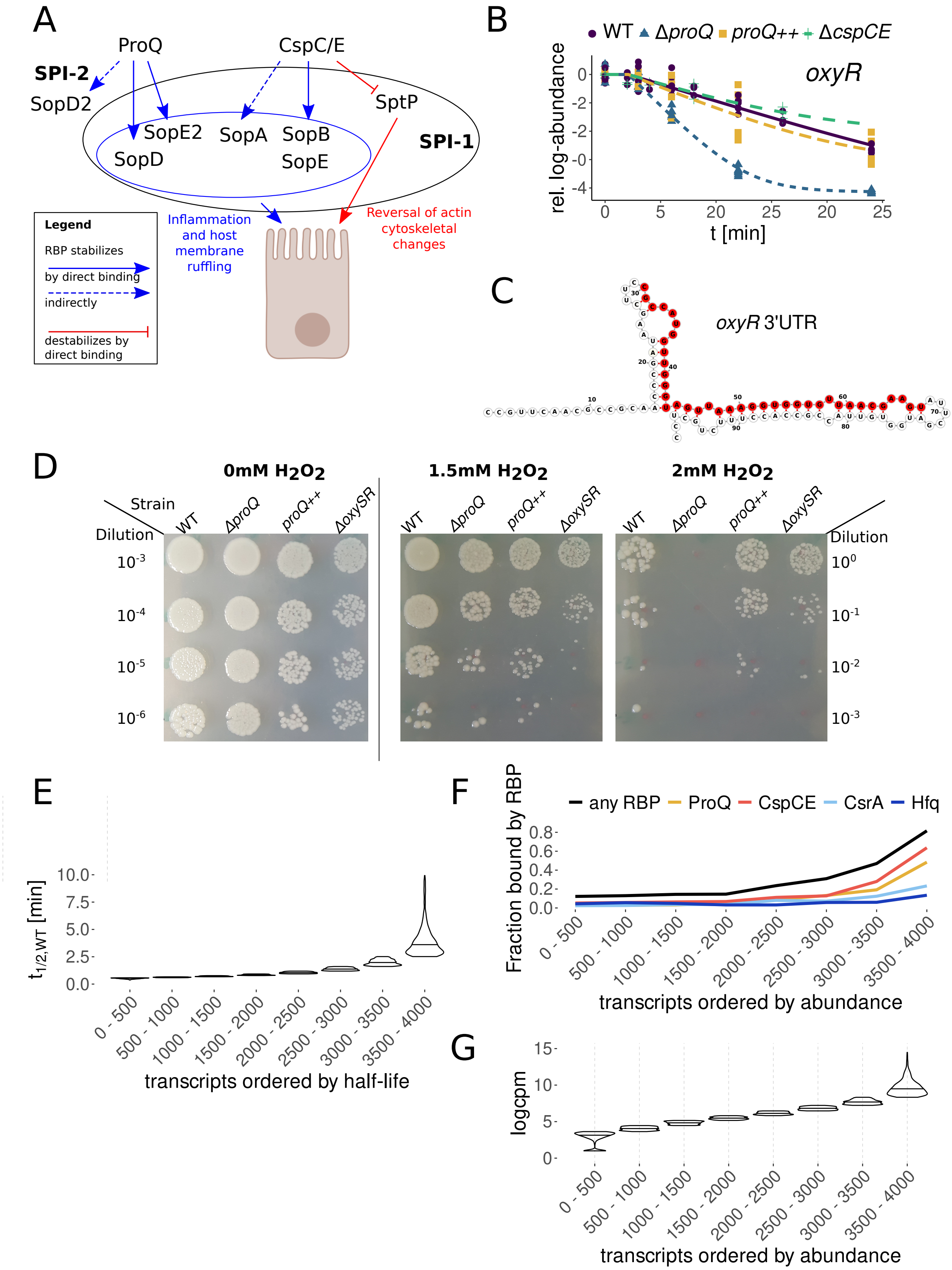
A
ProQ
CspC/E
SPI-2
SopD2
SopE2
SopA
SopB
SopD
SopE
SptP
SPI-1
Legend
RBP stabilizes
by direct binding
indirectly
destabilizes by direct binding
Inflammation and host membrane ruffling
Reversal of actin cytoskeletal changes
B
WT
ΔproQ
proQ++
ΔcspCE
oxyR
rel. log-abundance
t [min]
C
oxyR 3'UTR
D
0mM H2O2
1.5mM H2O2
2mM H2O2
Strain
Dilution
WT
ΔproQ
proQ++
ΔoxySR
E
t1/2,WT [min]
transcripts ordered by half-life
F
Fraction bound by RBP
any RBP
ProQ
CspCE
CsrA
Hfq
transcripts ordered by abundance
G
logcpm
transcripts ordered by abundance

Figure S9. Integrative analysis of RBP binding and transcript stability
(A) Regulation of SPI-1/2 effectors by ProQ and CspC/E. The picture of the epithelial cell was taken from BioRender.com. (B) Decay curve of significantly destabilized transcript of oxyR. (C) ProQ CLIP-seq peak in the 3'UTR of oxyR identified by re-analyzing (14), FDR=0.047. (D) Exposure of various Salmonella strains to varying levels of hydrogen peroxide. (E) WT half-life distributions for the same groups of transcripts as in Figure 5A. (F) Abundance dependence of CLIP-seq results. (G) Distribution of log-counts for the groups of transcripts used in (F).

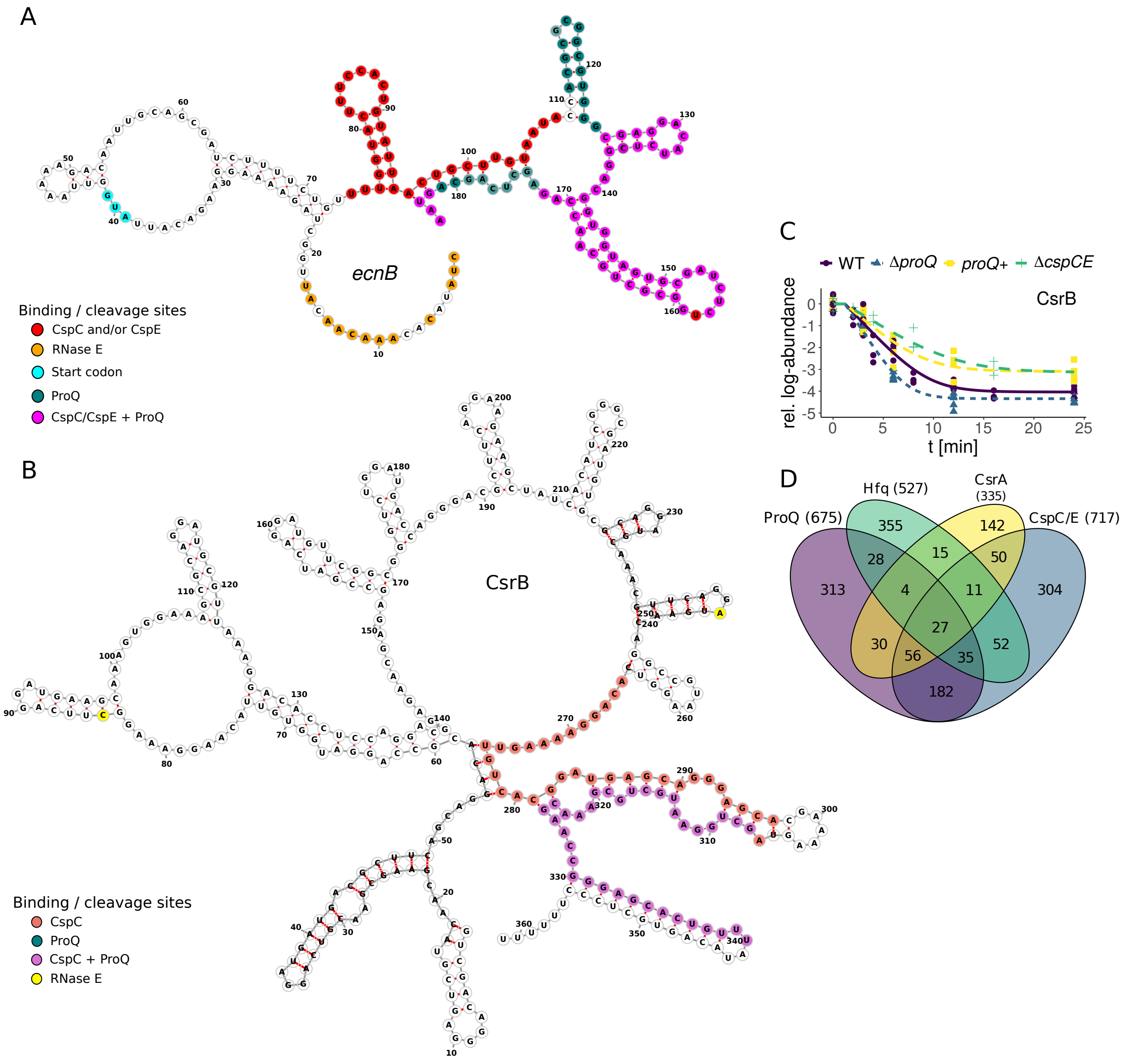

Figure S10. Integrative analysis of RNA secondary structures
(A) Secondary structure of the ProQ/CspC/E-bound mRNA of the bacteriolytic lipoprotein EcnB including RNase E cleavage sites (47). (B) Secondary structure of the ProQ/CspC/E-bound sRNA CsrB including RNase E cleavage sites (47). (C) Normalized data for CsrB, including the fitted decay curves. (D) Overlap in interaction partners between various CLIP-seq data sets with major RBPs.

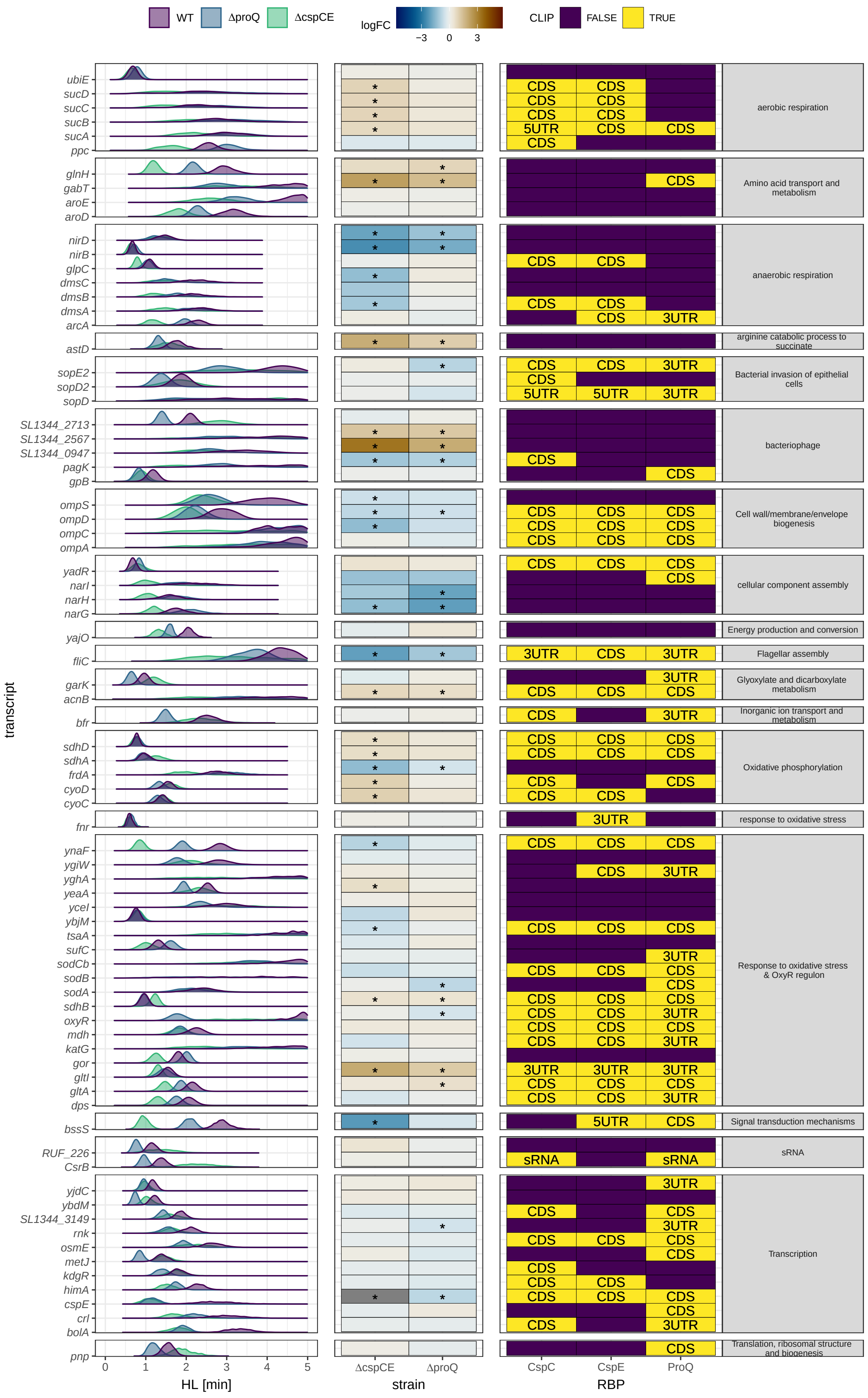

Figure S11. Top destabilized transcripts in the absence of ProQ and oxidative stress response

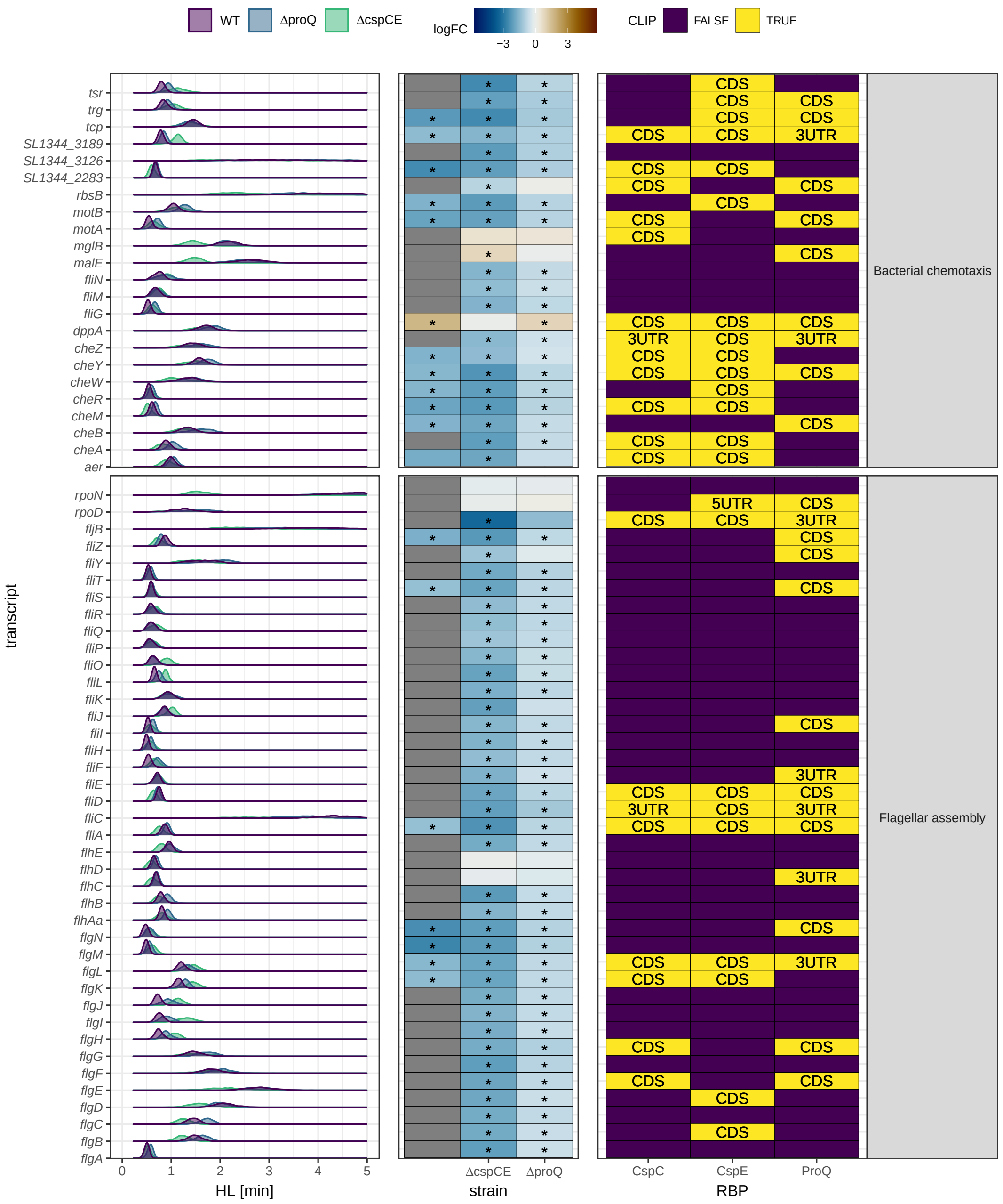

Figure S12. Flagellar genes with negative log-fold change in proQ and cspC/E deletion strains

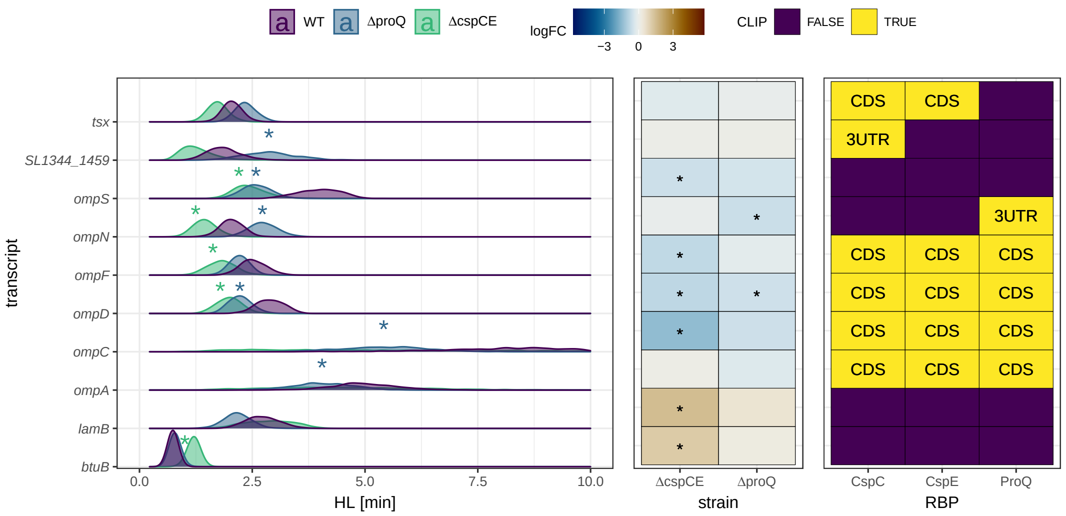

Figure S13. GO pathway porin activity
Significant changes are marked with stars.

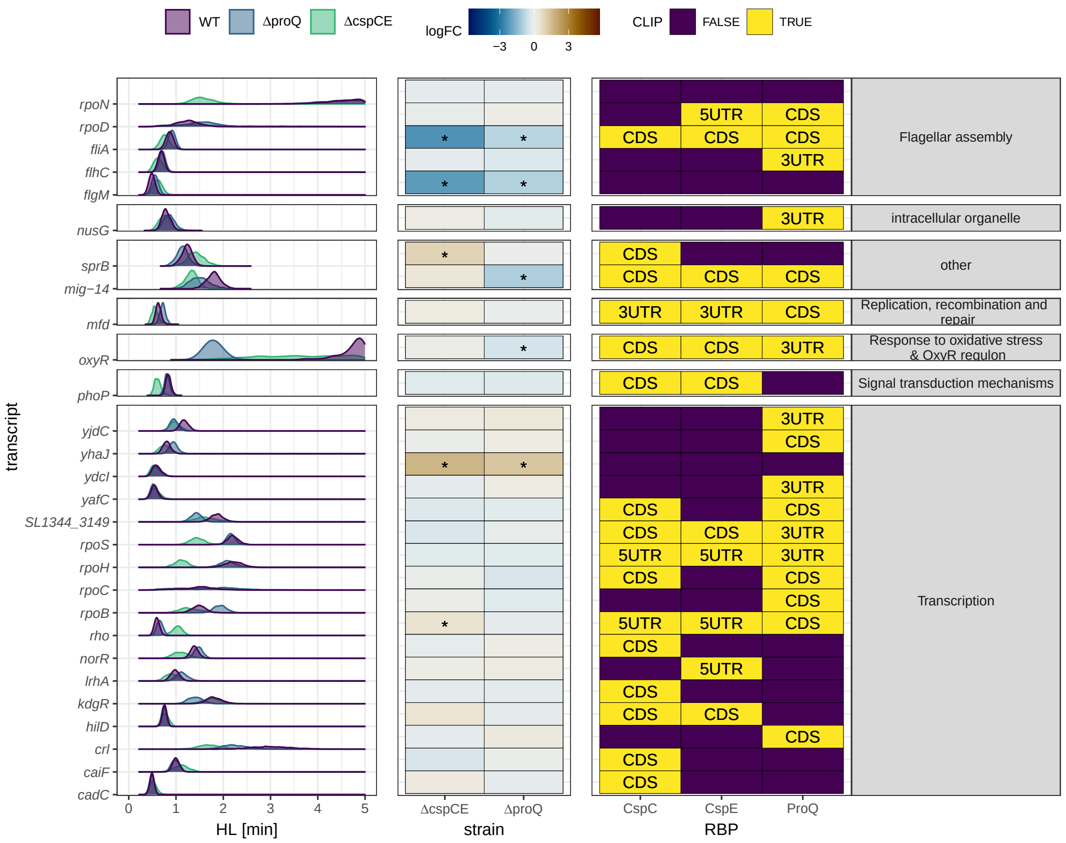

Figure S14. Figure S14. Transcription factors, subunits of RNA polymerase complex

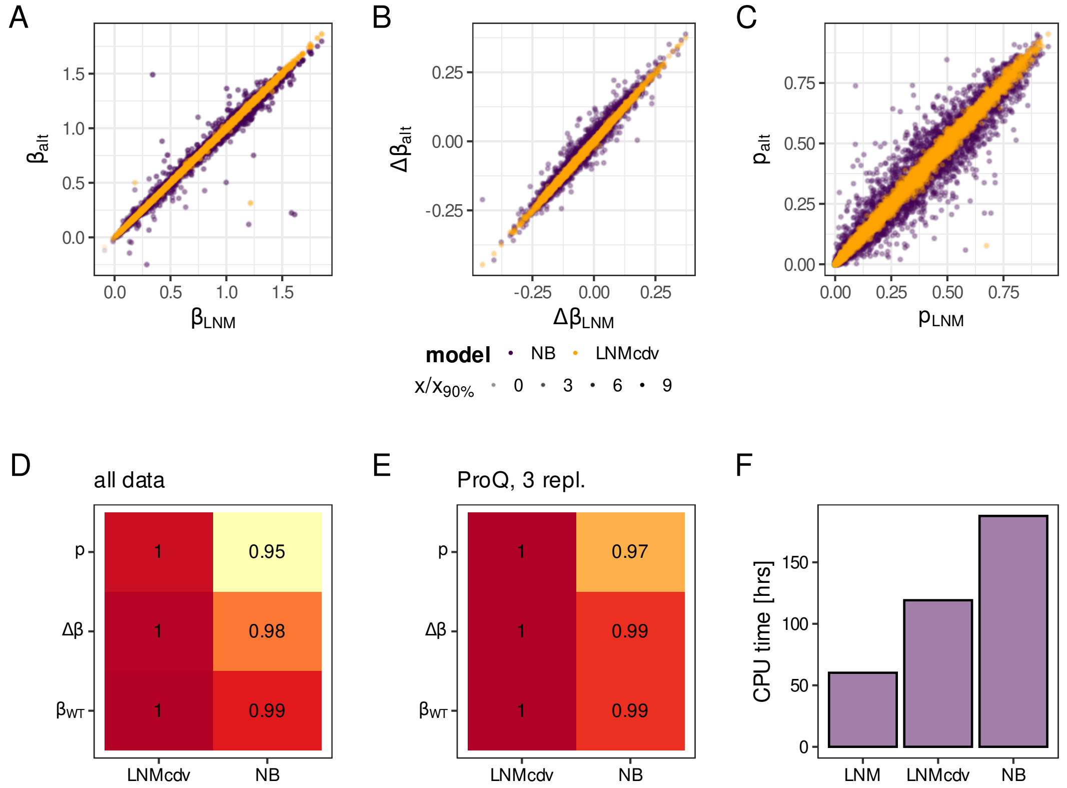

Figure S15. Figure S15. Comparison between count-based and log-normal models
(A) WT decay rate β, (B) difference in decay rate $\Delta\beta$, and (C) p-value in the LNM and two alternative models. (D) Heatmap of Spearman (p-value) and Pearson (decay rate/difference in decay rate) correlation coefficients between our LNM and two alternative models. The models were fitted to both the ProQ and the CspC/E RIF-seq datasets simultaneously. (E) Same as (D), but using only 3 replicates of the ProQ RIF-seq dataset. (F) CPU times required to fit the models. Times are given for 1 chain with 1,000 warmup and 1,000 sampling iterations.

Supporting Tables

trivial name	stock name	resistance marker	comments	reference
WT	JVS-1574	Str	Salmonella enterica sv. Typhimurium SL1344, Str-resistant hisG rpsL xyl	(18)
ΔproQ	JVS-10315	Str, Kan	Salmonella Typhimurium SL1344 proQ deletion strain created with the use of the lambda-Red system	(19)
ΔproQ	JVS-10317	Str	Salmonella Typhimurium SL1344 proQ deletion strain (JVS-10315) was cured with the use of the pCP20 plasmid	(19)
cspE-3xFLAG	JVS-4691			(20)
cspC-3xFLAG	JVS-4736			(20)
ΔcspCE::FRT	JVS-5084			(20)

Table S1. Bacterial strains used in this study.

trivial name	stock name	resistance marker	relevant insert	parental plasmid	reference
pZE-ctrl.	pJV-300	Amp	empty control plasmid	pZE12-luc	(21)
pZE12-ProQ	pZE12-ProQ	Amp	contains the proQ gene under control of its native promoter cloned into XbaI site in the same orientation as the plasmid-encoded terminator	pZE12-luc	(19)

Table S2. Plasmids used in this study.

name	target	purpose	nucleotide sequence	reference
JVO-14985	Salmonella proQ	sense oligo for cloning of proQ + own promoter into pXG10. with BfrBI site	gttttATGCATAATCAACGGATAACGTAGCAATTACT	(22)
JVO-14986	Salmonella proQ	antisense oligo for cloning of proQ + own promoter into pXG10. with NheI site	gttttGCTAGCGCCAGGCCTGGCCTCCG	(22)

JVO_35<br>87		Sense oligo for insertion of<br>3x-Flag downstream of cspC	CGGCCAGAAAGGTCCGG<br>CTGCTGTTAACGTAACAG<br>CTATCGACTACAAAGACCA<br>TGACGG	
JVO_35<br>88		Antisense oligo for insertion of<br>3x-Flag downstream of cspC.<br>Keeps endogenous rho<br>independent terminator	GAATTAAAAAGCCCCGCT<br>TTTAGCGAGGCTTTATATC<br>TGACCATATGAATATCCTC<br>CTTAG	
JVO_35<br>89		Sense oligo for verification of<br>CspC Flag tagging	ACACTTCTCCGCTATCCA	
JVO_35<br>90		Antisense oligo for verification of<br>CspC Flag tagging	GCAACAAATCGCTAACGA	
JVO_38<br>83		Sense oligo for insertion of<br>3xFlag to the last codon of cspE	ACTAACGGTGCCAAAGGC<br>CCTTCCGCTGCAAACGTA<br>ACTGCTCTGGACTACAAA<br>GACCATGACGG	
JVO_38<br>84		Antisense olig o for insertion of<br>3xFlag downstream of cspE,<br>keep the terminator	CGTCCGACAGCAAGATTT<br>C<br>AAAACCCGCCCTTTCGGC<br>GGGTTTTTCCATATGAATA<br>TCCTCCTTAG	
JVO_38<br>85		Sense oligo for verification of<br>CspE -3xFlag tagging	CTGCAATCCAGACCAATG	
JVO_38<br>86		Antisense oligo for verification of<br>CspE -3xFlag tagging	TTCGTTTGCCATGACG	
JVO_44<br>96		Forward oligo to delete cspC<br>CDS in Salmonella	atgcctacgggcaaagaaaacactc<br>taaggaattttgcaaGTGTAGGC<br>TGGAGCT	
JVO_44<br>97		Reverse oligo to delete cspC<br>CDS in Salmonella	tgaggctgacgcgacaaacgcatca<br>aatcagtggtgtcgaGGTCCATA<br>TGAATATCCTCCTTAG	
JVO_44<br>98		Forward oligo for verification of<br>cspC deletion in Salmonella	CCGAACCTTATATTAGTGC<br>C	
JVO_44<br>99		Reverse oligo for verification of<br>cspC deletion in Salmonella	CCAGACACATTTTGAAGG<br>ATA	

JVO_45 04		Forward oligo to delete cspE CDS in Salmonella	cacagcatttgtgtctatttttcatgtaaa ggtaattttgGTGTAGGCTGGA GC TGCTTC	
JVO_45 05		Reverse oligo to delete cspE CDS in Salmonella	cgaaagggcgggttttgaaatcttgct gtcggacgtatgcGGTCCATAT GAATA TCCTCCTTAG	
JVO_45 06		Forward oligo for verification of cspE deletion in Salmonella	GACTGGACAAAATGCATC AC	
JVO_45 07		Reverse oligo for verification of cspE deletion in Salmonella	GCTGAATGTGCTGATTAAC C	

Table S3. Oligos used in this study.

type	name	target	source	dilution	provider
primary	anti-FLAG	FLAG epitope	mouse	1:1000 (in 3% BSA)	Sigma

Table S4. Antibodies used in this study.

Supporting References

1. A. R. Gruber, R. Lorenz, S. H. Bernhart, R. Neuböck, I. L. Hofacker, The Vienna RNA websuite. *Nucleic Acids Res.* **36**, W70–4 (2008).

2. P. Kerpedjiev, S. Hammer, I. L. Hofacker, Forna (force-directed RNA): Simple and effective online RNA secondary structure diagrams. *Bioinformatics* **31**, 3377–3379 (2015).

3. J. Huerta-Cepas, *et al.*, eggNOG 5.0: a hierarchical, functionally and phylogenetically annotated orthology resource based on 5090 organisms and 2502 viruses. *Nucleic Acids Res.* **47**, D309–D314 (2019).

4. D. Binns, *et al.*, QuickGO: a web-based tool for Gene Ontology searching. *Bioinformatics* **25**, 3045–3046 (2009).

5. M. Kanehisa, Y. Sato, M. Kawashima, M. Furumichi, M. Tanabe, KEGG as a reference resource for gene and protein annotation. *Nucleic Acids Res.* **44**, D457–62 (2016).

6. A. Subramanian, *et al.*, Gene set enrichment analysis: a knowledge-based approach for interpreting genome-wide expression profiles. *Proc. Natl. Acad. Sci. U. S. A.* **102**, 15545–15550 (2005).

7. Y. Chao, *et al.*, In Vivo Cleavage Map Illuminates the Central Role of RNase E in Coding and Non-coding RNA Pathways. *Mol. Cell* **65**, 39–51 (2017).

8. E. Holmqvist, *et al.*, Global RNA recognition patterns of post-transcriptional regulators Hfq and CsrA revealed by UV crosslinking in vivo. *EMBO J.* **35**, 991–1011 (2016).

9. E. Holmqvist, L. Li, T. Bischler, L. Barquist, J. Vogel, Global Maps of ProQ Binding In Vivo Reveal Target Recognition via RNA Structure and Stability Control at mRNA 3' Ends. *Mol. Cell* **70**, 971–982.e6 (2018).

10. H. Xu, *et al.*, FastUniq: a fast de novo duplicates removal tool for paired short reads. *PLoS One* **7**, e52249 (2012).

11. M. Martin, Cutadapt removes adapter sequences from high-throughput sequencing reads. *EMBnet.journal* **17**, 10–12 (2011).

12. S. Hoffmann, *et al.*, A multi-split mapping algorithm for circular RNA, splicing, trans-splicing and fusion detection. *Genome Biol.* **15**, R34 (2014).

13. A. Dobin, *et al.*, STAR: ultrafast universal RNA-seq aligner. *Bioinformatics* **29**, 15–21 (2013).

14. M. I. Love, W. Huber, S. Anders, Moderated estimation of fold change and dispersion for RNA-seq data with DESeq2. *Genome Biol.* **15**, 550 (2014).

15. A. Zhu, J. G. Ibrahim, M. I. Love, Heavy-tailed prior distributions for sequence count data: removing the noise and preserving large differences. *Bioinformatics* **35**, 2084–2092 (2018).

16. M. D. Robinson, D. J. McCarthy, G. K. Smyth, edgeR: a Bioconductor package for differential expression analysis of digital gene expression data. *Bioinformatics* **26**, 139–140 (2010).

17. C. W. Law, Y. Chen, W. Shi, G. K. Smyth, voom: Precision weights unlock linear model analysis tools for RNA-seq read counts. *Genome Biol.* **15**, R29 (2014).

18. S. K. Hoiseth, B. A. Stocker, Aromatic-dependent Salmonella typhimurium are non-virulent and effective as live vaccines. *Nature* **291**, 238–239 (1981).

19. A. Smirnov, *et al.*, Grad-seq guides the discovery of ProQ as a major small RNA-binding protein. *Proc. Natl. Acad. Sci. U. S. A.* **113**, 11591–11596 (2016).

20. C. Michaux, *et al.*, RNA target profiles direct the discovery of virulence functions for the cold-shock proteins CspC and CspE. *Proc. Natl. Acad. Sci. U. S. A.*, 201620772 (2017).

21. J. H. Urban, J. Vogel, Translational control and target recognition by Escherichia coli small RNAs in vivo. *Nucleic Acids Res.* **35**, 1018–1037 (2007).

22. A. J. Westermann, *et al.*, The Major RNA-Binding Protein ProQ Impacts Virulence Gene Expression in Salmonella enterica Serovar Typhimurium. *MBio* **10** (2019).
